# Amnestic Mild Cognitive Impairment Is Associated With Frequency-Specific Brain Network Alterations in Temporal Poles

**DOI:** 10.3389/fnagi.2018.00400

**Published:** 2018-12-06

**Authors:** Francesca Jacini, Pierpaolo Sorrentino, Anna Lardone, Rosaria Rucco, Fabio Baselice, Carlo Cavaliere, Marco Aiello, Mario Orsini, Alessandro Iavarone, Valentino Manzo, Anna Carotenuto, Carmine Granata, Arjan Hillebrand, Giuseppe Sorrentino

**Affiliations:** ^1^Department of Motor Sciences and Wellness, Parthenope University of Naples, Naples, Italy; ^2^Institute for Diagnosis and Cure Hermitage Capodimonte, Naples, Italy; ^3^Department of Engineering, Parthenope University of Naples, Naples, Italy; ^4^Department of Clinical Neurophysiology and MEG Center, VU University Medical Center Amsterdam, Amsterdam, Netherlands; ^5^Diagnostic and Nuclear Research Institute, IRCCS SDN, Naples, Italy; ^6^Neurological and Stroke Unit, CTO Hospital—AORN Ospedale dei Colli, Naples, Italy; ^7^Department of Neurology, AORN Cardarelli, Naples, Italy; ^8^Institute of Applied Sciences and Intelligent Systems, CNR, Pozzuoli, Italy

**Keywords:** Alzheimer’s disease (AD), mild cognitive impairment—MCI, network analysis, magnetoencephalography (MEG), phase lag index (PLI), minimum spanning tree (MST), functional connectivity (FC)

## Abstract

There is general agreement that the neuropathological processes leading to Alzheimer’s disease (AD) begin decades before the clinical onset. In order to detect early topological changes, we applied functional connectivity and network analysis to magnetoencephalographic (MEG) data obtained from 16 patients with amnestic Mild Cognitive Impairment (aMCI), a prodromal stage of AD, and 16 matched healthy control (HCs). Significant differences between the two groups were found in the theta band, which is associated with memory processes, in both temporal poles (TPs). In aMCI, the degree and betweenness centrality (BC) were lower in the left superior TP, whereas in the right middle TP the BC was higher. A statistically significant negative linear correlation was found between the BC of the left superior TP and a delayed recall score, a sensitive marker of the “hippocampal memory” deficit in early AD. Our results suggest that the TPs, which are involved early in AD pathology and belong to the memory circuitry, have an altered role in the functional network in aMCI.

## Introduction

A large number of studies have investigated the causes of Alzheimer’s disease (AD); nevertheless, many uncertainties remain about its pathophysiology (Kanfer et al., 1999; Sorrentino et al., 2014). It is likely that multiple mechanisms contribute to the deposits of senile plaques and neurofibrillary tangles, the hallmarks of AD pathology (Sorrentino et al., 2008). It is well established that the spatiotemporal pattern of progression of neurofibrillary degeneration starts in the allocortex of the medial temporal lobe (entorhinal cortex and hippocampus) and spreads to the associative neocortex (Braak and Braak, 1991; Braak et al., 2006). Furthermore, it is now a general understanding that the neuropathological processes leading to AD begin decades before the onset of clinical symptoms (Sorrentino and Bonavita, 2007; Jack et al., 2010).

Mild Cognitive Impairment (MCI) has been characterized by an objective cognitive impairment in a given domain, mainly memory, not yet implying the definition of dementia (Petersen et al., 1999). Yet, MCI is associated with a higher risk of developing dementia, including AD (Shah et al., 2000; Petersen et al., 2009). In recent years, this definition underwent significant evolution. The original notion of a predominant amnestic problem was substituted by a new perspective where different clinical subtypes were defined. Nowadays, MCI patients are categorized according to type and number of affected cognitive domains. This clinical classification is particularly relevant because each subtype is linked to a presumed etiology, with the amnestic subtypes (aMCI) considered as a prodromal form of AD (Petersen et al., 2009, 2014).

The human brain can be seen as a complex system characterized by a balancing between integration and segregation mechanisms through spatial and temporal interaction of distinct neuronal populations (Sporns et al., 2005; Lopes da Silva, 2013; Sporns, 2013). One of the strategies to estimate the interactions between brain areas, referred to as “functional connectivity” (Friston, 2011), is to exploit statistical dependencies that might be present between the time series of neuronal activation in these areas. Brain networks can be characterized using a combination of graph theory and modern network science, applied to neuroimaging data obtained from techniques such as functional magnetic resonance imaging (fMRI; Stam, 2010; Wang, 2010). To enable characterization of the macroscopic brain network topology using graph analysis, brain areas are typically used as nodes and the interactions between the brain areas as links.

It has been proposed that in neurodegenerative diseases, the spreading of the pathological process reflects itself in the reduction of structural and functional connectivity (Trojsi et al., 2017), and it has been suggested that AD may be regarded as a disconnection syndrome (Pievani et al., 2011; Minati et al., 2013; Stam, 2014). Previous studies showed that the network architecture of AD brains loses the typical “small world” organization (Watts and Strogatz, 1998), disrupting the balance between functionally highly specialized areas and long-range interactions between distant regions (Stam et al., 2009; Sanz-Arigita et al., 2010; Stam, 2010). There is also some evidence that the most connected nodes are preferentially affected by pathological processes (Buckner et al., 2009; Stam, 2014) perhaps due to their high metabolic activity (de Haan et al., 2012). Starting from there, the functional disconnection might spread to other brain regions (Brier et al., 2014). Possibly, damage to a node could lead to overload, and subsequent failure, of the hierarchically upstream nodes. This could be the mechanism underlying the spreading of neurodegeneration (Stam, 2014). Hence, in order to capture the subtle changes underlying cognitive impairment, we need to evaluate the brain as a complex system (Sporns, 2011).

There is ample evidence that a temporally and spatially balanced pattern of synchronized and desynchronized oscillations in distinct frequency bands underlies specific brain functions, including cognitive functions such as language, memory, thought or awareness (Gross et al., 2004). Beside fMRI, relevant information on the functioning of brain networks can be retrieved using neurophysiological techniques, such as electroencephalography (EEG) and magnetoencephalography (MEG), since they directly capture the oscillatory activity of the neuronal ensembles, thereby providing clinical and pathophysiological information about brain functioning in health and disease (Lopes da Silva, 2013). While retaining the high temporal resolution of EEG, MEG signals are not distorted by the layers surrounding the brain, allowing for a temporally and spatially precise reconstruction of the neural activity within the brain (Baillet, 2017). Furthermore, the high temporal resolution of MEG signals allows for a more sophisticated estimation of synchronization between brain areas. For example, the phase lag index (PLI; Stam et al., 2007) is a phase-based metric (insensitive to volume conduction/field spread) that quantifies phase-synchronization (Rosenblum et al., 1996) between areas, rather than the simultaneous amplitude fluctuations typically estimated with fMRI.

Several MEG studies have addressed network changes in AD, but the results have not always been consistent (for an extensive review see Engels et al., 2017). The reason for the non-homogeneity of findings may have its origin in both the clinical features of the examined populations and the analysis methods. AD has a long clinical course linked to a well-defined neuropathological evolution (Braak and Braak, 1991, 1995, 1996). As a consequence, the disease stage at which the recording takes place may be crucial. Another possible source of inconsistency of the experimental observations may lie in the fact that network reconstructions may be biased (van Wijk et al., 2010). In particular, one main problem is the comparison of networks with different edge densities, average degrees, or edge weights. Normalization of the data or selection of arbitrary cut-offs does not solve this issue (van Wijk et al., 2010; Otte et al., 2015). A possible approach to overcome these limitations is the use of the minimum spanning tree (MST; Stam et al., 2014). Starting from the full network, the MST allows for the construction of a unique sub-graph that connects all the nodes without forming cycles. This way, it is possible to identify the backbone of the original network. If the link weights are unique, this procedure provides a unique reconstruction of networks with the same number of nodes and links. The metrics calculated on the MST capture information of the original network (Tewarie et al., 2015) while allowing the unbiased statistical comparisons of those metrics across groups, in the sense that observed topological differences are not trivially due to differences in functional connectivity (van Wijk et al., 2010).

The aim of our study is to identify subtle changes in network topology in aMCI. Given that aMCI is considered a prodromal stage of AD, our hypothesis is that in aMCI the topological alterations of the network involve brain areas that are known to degenerate early in AD. Furthermore, we hypothesized that such alterations might be specific to brain rhythms related to memory processes. Detecting subtle functional alterations might enable the identification of early disease markers and to shed light on the pathophysiological mechanisms of neurodegeneration. To test our hypothesis, we applied the PLI, followed by the MST, to MEG data obtained from a cohort of aMCI patients and healthy controls (HCs), and compared the reconstructed brain networks between these groups.

## Materials and Methods

### Participants

Fifty-two participants, aged 65–80 years, were screened. All subjects were right handed and native Italian speakers. Exclusion criteria were the presence of neurological or systemic illness that could affect the cognitive status, and contraindications to MRI or MEG recording. Based on neurological examination and extensive neuropsychological assessment (see Table 1), subjects were divided into two groups: 21 MCI and 31 controls. MCI diagnosis was formulated according to the National Institute on Aging-Alzheimer Association (NIA-AA) criteria (Albert et al., 2011), which include: (i) cognitive concern reported by patient or informant or clinician; (ii) objective evidence of impairment in one or more cognitive domains, typically including memory; (iii) preservation of independence in functional abilities; and (iv) not demented. Three of the MCI patients were non-amnestic MCI so they were excluded from the study. Reduced hippocampal volume detected by structural MRI (see Table 2), a neuronal injury marker, gives our MCI cohort an intermediate likelihood of being due to AD (Albert et al., 2011). Two patients and 15 controls did not complete the MRI because of difficulty in lying down or refusal to perform it, so they were excluded.

**Table 1:** Neuropsychological evaluation.

Test	Explored function
**MMSE**	Global cognitive status
**FAB**	Frontal efficiency
**FCSRT**	
FCSRT immediate free recall	
FCSRT immediate total recall	“Hippocampal” episodic memory
FCSRT delayed free recall	
FCSRT delayed total recall	
FCSRT index of sensitivity of cueing	
**MDB**	
Rey’s 15 word immediate recall	Short and long-term verbal
Rey’s 15 word delayed recall	episodic memory
Word fluency	Ability to access lexical-semantic memory store
Phrase construction	Language
Raven’s 47 progressive matrices	Conceptual reasoning
Immediate visual memory	Short-term visuoperceptual recognition memory
Freehand copying of drawings	Constructive praxia
Copying drawings with landmarks	
**BDI**	Depression

*MMSE, Mini Mental State Examination (Measso et al., 1993); FAB, Frontal Assessment Battery (Iavarone et al., 2004); FCSRT, Free and Cued Selective Reminding Test (Frasson et al., 2011); MDB, Mental Deterioration Battery (Carlesimo et al., 1996); BDI, Beck Depression Inventory (Sica and Ghisi, 2007)*.

**Table 2:** Subjects characteristics.

	aMCI (*n* = 16)	HC (*n* = 16)	*p*-value
Age (years)	73 ± 6.6	70 ± 3.9	NS
Education (years)	10.25 ± 4.55	13 ± 4.81	NS
Left hippocampal volume (%)	0.230 ± 0.039	0.288 ± 0.043	<0.001
Right hippocampal volume (%)	0.223 ± 0.030	0.285 ± 0.036	<0.001
MMSE	25.00 ± 2.62	28.21 ± 1.42	0.002
FCSRT immediate free recall	15.95 ± 7.60	28.21 ± 1.42	<0.001
FCSRT delayed free recall	3.57 ± 3.42	10.47 ± 1.53	<0.001
FCSRT immediate total recall	28.93 ± 6.45	35.93 ± 0.25	<0.001
FCSRT delayed total recall	7.68 ± 3.32	12 ± 0	<0.001
FCSRT index of sensitivity	0.70 ± 0.20	0.99 ± 0.02	<0.001
BDI	9.6 ± 8	7.1 ± 4.9	NS
FAB	14.35 ± 2.80	16.44 ± 1.19	0.010
Rey’s 15 word immediate recall	26.27 ± 5.98	44.23 ± 7.76	<0.001
Rey’s 15 word delayed recall	3.56 ± 1.89	10.53 ± 3.21	<0.001
Word fluency	32.87 ± 11.29	32.71 ± 6.49	NS
Phrase construction	14.56 ± 8.15	18.14 ± 5.67	NS
Raven’s 47 progressive matrices	22.3 ± 5.65	28.03 ± 4.34	0.003
Immediate visual memory	16.10 ± 3.77	19.87 ± 1.88	0.001
Freehand copying of drawings	8.41 ± 1.36	9.98 ± 1.38	0.003
Copying drawings with landmarks	65.57 ± 3.78	68.58 ± 4.58	NS

*Demographic and clinical variables comparison in aMCI and control groups. Data are given as mean ± standard deviation (SD). NS, not significant; aMCI, amnestic Mild Cognitive Impairment group; HC, healthy controls; MMSE, Mini Mental State Examination; FCSRT, Free and Cued Selective Reminding Test; BDI, Beck Depression Inventory; FAB, Frontal Assessment Battery*.

The subjects included in the study were 16 patients affected by aMCI (mean age 71, 7 years; standard deviation (SD). 6, 6; eight men and eight women) compared to 16 age, educational level and gender matched HC subjects (mean age 70, 3 years; SD 4, 2; nine man and seven women). The study was approved by the Local Ethics Committee “Comitato Etico Campania Centro” (Prot.n.93C.E./Reg. n.14-17OSS), and all subjects had given written informed consent. All methods included in the protocol were carried out in accordance with the Declaration of Helsinki.

### Magnetic Resonance Imaging

MR images were acquired using a 3T Biograph mMR tomograph (Siemens Healthcare, Erlangen, Germany) equipped with a 12 channels head coil. The scan performed either after the MEG recording or a minimum of 21 days earlier (within 1 month). The following protocol was applied: (i) three-dimensional T1-weighted Magnetization-Prepared Rapid Acquisition Gradient-Echo sequence (MPRAGE, 240 sagittal planes, 214 × 21 mm^2^ Field of View, voxel size 1 × 1 × 1 mm^3^, TR/TE/TI 2,400/2.5/1,000 ms, flip angle 8°); (ii) Three-dimensional T2-weighted Sampling Perfection with Application optimized Contrasts using different flip angle Evolution sequence (SPACE, 240 sagittal planes, 214 × 214 mm^2^ Field of View, voxel size 1 × 1 × 1 mm^3^, TR/TE 3,370/563); (iii) Two-dimensional T2-weighted turbo spin echo Fluid Attenuated Inversion Recovery sequence (FLAIR, 44 axial planes, 230 × 230 mm^2^ Field of View, voxel size 0.9 × 0.9 × 0.9 mm^3^, TR/TE/TI 9,000/95/25,00, flip angle 150°). Volumetric analysis was performed with the Freesurfer software (version 6.0; Fischl et al., 2002). Volumes were normalized for the estimated total intracranial volume (eTIV). Vascular burden was assessed using Fazekas scale (Fazekas et al., 1987).

### MEG Acquisition

The MEG system, equipped with 163 magnetometers, was developed by the National Research Council, Pozzuoli, Naples, at the Institute of Applied Sciences and Intelligent Systems “E. Caianiello” (Rombetto et al., 2014). Using Polhemus (Polhemus FASTRAK^®^) we determined the location of four coils, placed on the forehead and behind the ears of the participants, and of four reference points on the head (nasion, right and left preauricular points, vertex). The head movement were evaluated by visual inspection through a camera placed inside the cabin. The coils were activated, and localized, at the beginning of each segment of the registration. Participants were seated inside a magnetically shielded room to reduce background noise (Advanced Technologies Biomagnetics, Ulm, Germany). Electrocardiographic (ECG) and Electrooculographic (EOG) signals were co-recorded to aid artifact removal (Gross et al., 2013). Spontaneous brain activity was recorded for two sets of 2.5 min, in resting-state, with eyes closed. Signals, after an anti-aliasing filter, were acquired at a sampling frequency of 1,024 Hz. The signal was then filtered using a fourth order Butterworth IIR band-pass filter in the 0.5–48 Hz band. In order to reduce the environmental noise, principal component analysis (PCA) was implemented (Sadasivan and Dutt, 1996). The methodology consists in computing the space base of the signals acquired by the reference sensors (i.e., environmental noise), and in projecting the signals from the brain sensors on such base in order to remove the noise parallel components from the brain signal (de Cheveigné and Simon, 2007). The PCA filtering implementation available within the Fieldtrip Toolbox (Oostenveld et al., 2011) was used. Subsequently, noisy channels (on average 14 ± 5 channels) were removed manually through visual inspection of the whole dataset by an experienced rater (Gross et al., 2013). Physiological artifacts, cardiac and blinking (if present), were removed from the signals through supervised independent component analysis (ICA). Typically, one component has been deleted for ECG and no component for EOG. The processing has been done using the Fieldtrip toolbox (version 2014.05.06; Oostenveld et al., 2011).

### Source Reconstruction

The subject’s fiducial points were visually identified on the native MRI of the subjects and used to coregister the MEG acquisition, whereupon the MRI was spatially normalized to an average T1-MRI template, part of the SPM toolbox^1^. Subsequently, we used the volume conduction model proposed by Nolte (2003) and we applied the Linearly Constrained Minimum Variance (LCMV) beamformer (Van Veen et al., 1997) to reconstruct the time series in the centroids of 116 regions-of-interest (ROIs). In order to compute the beamformer weights, we used the broad-band data covariance matrix and an identity noise covariance matrix (hence assuming uncorrelated noise). The optimal source orientation was found using a Singular Value Decomposition (SVD). The labeling of the regions was based upon the Automated Anatomical Labeling (AAL) atlas (Tzourio-Mazoyer et al., 2002; Gong et al., 2009). We considered only the first 90 ROIs, excluding those corresponding to the cerebellum given the low reliability of the reconstructed signal in those areas. For each region, we projected the time series along the dipole direction that explained most variance by mean of SVD using Fieldtrip.

The signals in the source space have been downsampled to 512 Hz. By visual inspection, the first 10 epochs of 8 s for each subject that did not contain artifacts (either system related or physiological) or strong environmental noise were selected. All the source reconstruction processing has been performed in Matlab environment using the Fieldtrip toolbox.

### Functional Connectivity Analysis

The functional connectivity analysis was performed using BrainWave software (version 0.9.152.4.1, available from https://home.kpn.nl/stam7883/brainwave.html). The length of 8 s is a trade-off between the need to have enough cleaned epochs (Gross et al., 2013; Sorrentino et al., 2017) and to obtain a reliable estimate of the functional connectivity (Fraschini et al., 2014), while avoiding drowsiness. The epochs were band-pass filtered into five canonical frequency bands using Brainwave: delta (0.5–4 Hz), theta (4–8 Hz), alpha (8–13 Hz), beta (13–30 Hz) and gamma (30–48 Hz). The PLI (Stam et al., 2007) was used to estimate functional connectivity. The PLI is based on the distribution of the differences of the instantaneous phases (derived from the Hilbert transformation of the times series) for two time series, and is computed as:

$$PLI=\left|\langle sign\left[\sin\left(\text{ΔΦ}(t_{\text{k}})\right)\right]\rangle \right|$$

where “< >” indicates the mean value, “sign” stands the *signum* function, “|.|” denotes the absolute value and “*t*_k_” are the samples. This measure is not sensitive to volume conduction (at the cost of discarding true zero–lag interactions). PLI values range between 0 and 1, where 1 indicates perfect synchronization and 0 indicates non synchronous activity. We obtained a 90 × 90 adjacency matrix for each epoch for each subject, in all the frequency bands (Stam et al., 2007). For each epoch, the PLI matrix was computed, and after this step they are merged by arithmetic average.

### Network Analysis

The adjacency matrices we obtained were interpreted as networks, where the 90 sources are the nodes and the 1/PLI values are the edges. For each frequency band, a MST was calculated based on each adjacency matrix using Kruskal’s algorithm (Kruskal, 1956). The algorithm classifies the links in growing order and, then, builds the network by adding a link at a time. If the link forms a loop, it is discarded. The algorithm proceeds until all nodes are connected resulting in a loop-less binary graph with *N* nodes and *M* = *N* − 1 links. The MST was used to obtain topological measures that are unaffected by degree distribution, matrix density or arbitrary thresholds (van Wijk et al., 2010).

**Figure 1:**
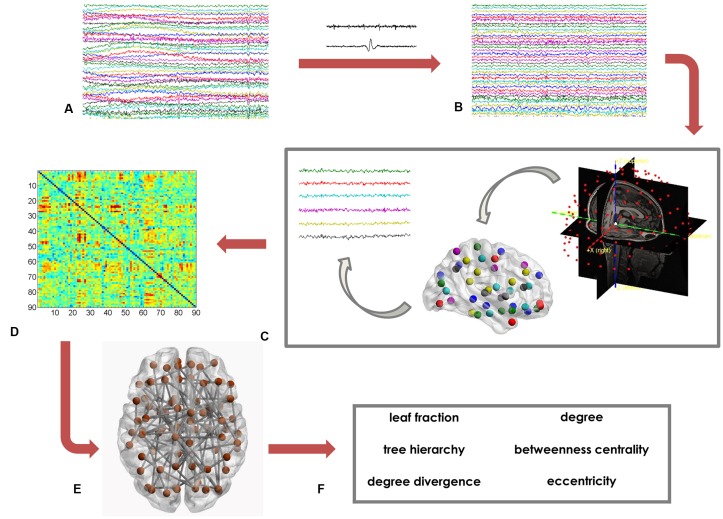
Data analysis pipeline. **(A)** Raw magnetoencephalography (MEG) signals recorded by 154 sensors (a subset displayed here). **(B)** MEG signals after artifact removal and noise cleaning. **(C)** Source reconstruction (beamforming), based on the native magnetic resonance imaging (MRI) of the subjects. **(D)** Functional connectivity matrix estimated using the phase lag index (PLI). Rows and columns are the regions of interest (ROIs), while the entries are the estimated values of the PLI. **(E)** Brain topology representation based on the minimum spanning tree (MST). **(F)** Global (leaf fraction, tree hierarchy and degree divergence) and nodal (degree, betweenness centrality (BC), eccentricity) network parameters.

Based on the MST matrix, we calculated both global and nodal parameters. The former provide insight on the network as a whole. In details, we calculated the leaf fraction (fraction of nodes with degree equal to 1), the tree hierarchy (quantification of the trade-off between node–overload and efficient communication), and the degree divergence (amplitude of the degree distribution; Boersma et al., 2013; Stam et al., 2014; Tewarie et al., 2015). Furthermore, we calculated nodal parameters that provide information on the centrality of each of the 90 ROI. We computed the degree, the betweenness centrality (BC) and the eccentricity. The degree is the number of connections incident on a given node. The BC represents the number of shortest paths passing through a given node, divided by the total number of shortest paths of the network (Boersma et al., 2013). The eccentricity is defined as the longest path between a node and any other node of the network. The lower the eccentricity, the more central the node (Tewarie et al., 2015). Figure 1 shows the data analysis pipeline (Sorrentino et al., 2018).

### Statistical Analysis

To compare clinical variables between the two groups we applied *T*-tests. MST metrics were compared, for each frequency band and for each parameter, between the two groups using permutation testing (N 10,000, alpha 0.05). In more detail: the labels of each metric (i.e., whether a value belongs to the aMCI or controls group) were randomly exchanged 10,000 times. At each iteration we computed the difference between the two groups, building a distribution of the differences. We used this distribution to compute the statistical significance of the experimentally observed groups difference. Then, the false discovery rate (FDR), using the Benjamini—Hochberg procedure (Benjamini and Hochberg, 1995), was applied to correct for multiple comparisons across the global MST measures (*N* = 3), and separately across the 90 ROIs for the nodal measures. The reported *p*-values that follow are always the FDR-corrected values (indicated as pFDR).

If a given node was found to have significantly different centrality in the two groups, we compared all the links incident upon that node in order to identify the ones that differed between the two groups, as follows: based on the PLI adjacency matrix, each link was averaged separately for the MCI and control groups, and the average strength was subsequently compared using permutation analysis, as described above. The results were corrected for multiple comparisons across links using FDR. To investigate the topological role of the links incident upon the significantly different nodes, we also checked how many times each link was included in the MST in both MCI and controls. To check if the frequency of inclusion in the MST differed between the two groups, we used a Chi-square test and corrected the results across the links using the FDR.

Finally, to calculate the correlation between clinical variables (neuropsychological scores) and both MRI data (hippocampal volume) and statistically significant network parameters, we used Pearson’s correlation coefficients. Correlations were calculated for the aMCI group only, because the neuropsychological scores in the control group showed a too narrow distribution to allow a reliable estimation of the correlation in this group. The linear correlations that have been computed were not corrected for multiple comparisons.

*T*-test and Pearson’s correlation coefficients were evaluated using IBM^®^ SPSS Statistics^®^ (version 20). Permutation testing and FDR correction were performed in Matlab (Mathworks^®^, version R2013a). A significance level of *p* < 0.05 was used.

## Results

### Population Characteristics

The studied population consists of 16 aMCI patients and 16 HC subjects. Comparing the clinical variables between the two groups, no significant differences were found in age, educational level, gender, depression (Beck Depression Inventory, BDI), language skills (fluency, phrase construction). Significant differences in hippocampal volume, global cognitive status (Mini Mental State Examination, MMSE), memory tests (Free and Cued Selective Reminding Test (FCSRT), Rey words, immediate visual memory), frontal efficiency (Frontal Assessment Battery, FAB) and constructive praxia (freehand copying of drawings, copying drawings with landmarks) were evident. As expected, patients showed worse cognitive performances (Table 2).

### MEG Data

**Figure 2:**
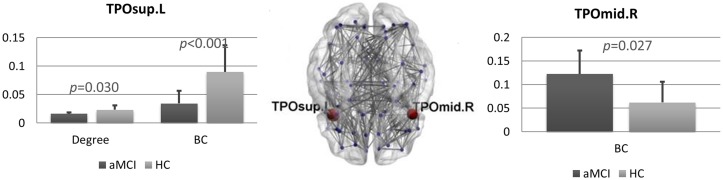
Brain networks comparison. Statistical differences between amnestic Mild Cognitive Impairment (aMCI) and control group in degree and BC in the theta band. Bar charts showing the mean degree and BC in the left superior temporal pole (TP; left) and mean BC in the right middle TP (right) for both groups. The *p*-values were computed using permutation testing (with false discovery rate (FDR) correction). In the middle, regional distribution of differences in degree and BC between aMCI and control group for the theta frequency band. MST network is visualized on a brain template. Blue dots represent the nodes of the network. In red, the nodes that are significantly different between aMCI and controls. Lines indicate the links between each pair of nodes. TPOsup.L, left superior temporal pole; TPOmid.R, right middle temporal pole.

We found several differences between the brain networks in aMCI and HCs. In the theta band, the degree (*pFDR* = 0.036) and the BC (*pFDR* < 0.001) of the left superior temporal pole (TP) were significantly lower in patients compared to controls. Conversely, in the right middle TP we found higher BC (*pFDR* = 0.027) in the aMCI group (Figure 2).

For the links incident upon the left superior TP, no individual link differed significantly between the two groups. The right medial TP, however, was more strongly connected to the right cuneus in controls as compared to the MCI population (*pFDR* = 0.0009).

No significant difference was found in the frequency of inclusion in the MST between the two groups for any link incident on the left superior or right middle TP.

The global MST parameters (leaf fraction, tree hierarchy and degree divergence) did not differ between the two groups. No statistically significant differences were found in any other frequency band (delta, alpha, beta and gamma) for any of the MST parameters.

### Correlations

**Figure 3:**
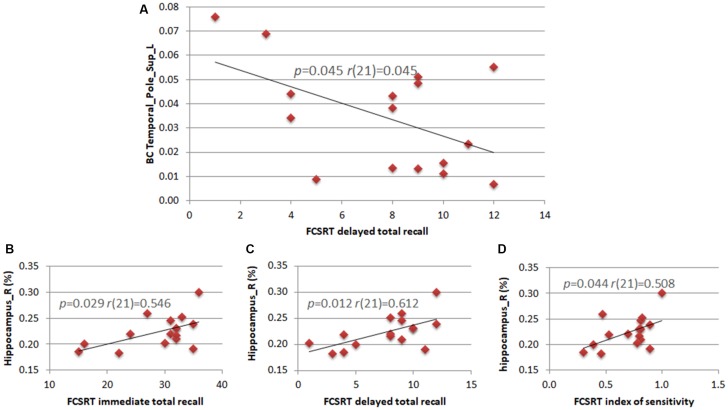
Correlations between neuropsychological scores, hippocampal volumes and network parameters. Pearson’s correlation between the BC of the left superior TP and the subtest of the Free and Cued Selective Reminding Test (FCSRT) regarding delayed total recall **(A)**. Pearson’s correlation between the right hippocampus volume and subtests of the FCSRT regarding immediate total recall **(B)**, delayed total recall **(C)** and index of sensitivity **(D)**.

We calculated the correlations between clinical variables (neuropsychological scores) and both MRI data (hippocampal volume) and statistically significant network parameters. The right hippocampal volume correlated positively with three of the five subtests of the FCSRT (immediate total recall (*r*_(21)_ = 0.546; *p* = 0.029), delayed total recall (*r*_(21)_ = 0.612; *p* = 0.012) and sensitivity index (*r*_(21)_ = 0.508; *p* = 0.044)). In theta band, the BC of the left superior TP correlated negatively with the FCSRT delayed total recall score (*r*_(21)_ = 0.507; *p* = 0.045; Figure 3).

## Discussion

In the present work, we aimed at identifying subtle changes in network topology in aMCI, which is considered as a prodromal stage of AD. Our hypothesis is that in aMCI the topological alterations of the functional network may be limited to brain regions typically affected by early neurodegeneration and to frequency bands involved in memory processing. To test our hypothesis, we applied the PLI followed by the MST on source-level MEG data obtained from a cohort of aMCI patients compared to HCs.

The two groups were comparable by age, educational level and depression status, while neuropsychological scores, especially memory tests and the hippocampal volume allowed a clear division between aMCI and controls. According to Albert’s criteria, given the presence of hippocampal atrophy, the likelihood that in our population the cognitive impairment was due to AD was “intermediate” (Albert et al., 2011). In addition, the observed positive correlation between right hippocampal volume and memory scores is in accordance with the role of hippocampal volume as a progression marker (Frankó et al., 2013). The right hippocampal volume directly correlated with three of the five subtests of the FCSRT (immediate total recall, delayed total recall, Index of Sensitivity of Cueing), confirming the strong link between hippocampal atrophy and memory efficiency (Stoub et al., 2010). It has been shown that the FCSRT test isolates storage disorders due to the involvement of medial temporal structures, that is typical of AD, from other memory deficits related to attention or retrieval disturbances (Frasson et al., 2011). This test has been included in the current diagnostic criteria for AD, since it detects specific episodic memory profiles characterized by low free recall score that is not normalized by cueing (Dubois et al., 2007, 2014). Correlation with the right hemisphere is consistent with the FCSRT visuospatial material used (de Toledo-Morrell et al., 2000) and confirms that our aMCI cohort displays clinical characteristics similar to the ones of the prodromal stage of AD (Dubois et al., 2014).

The global network parameters that we took into account (MST based leaf fraction, tree hierarchy and degree divergence) failed to show any difference between the two groups. AD is characterized by an overall decrease of functional connectivity, a loss of modularity and a specific vulnerability of long-range connections and of the hubs (Tijms et al., 2013). Generally, in MCI the network features might be considered as in an intermediate stage between AD and healthy subjects (Yao et al., 2010). Increased synchronization between brain areas has been demonstrated in MCI, especially for long-distance connections, as well as reduced modularity, with altered segregation/integration balance (Buldú et al., 2011). In contrast, reduced functional connectivity has recently been reported for medial temporal and parietal regions in MCI compared to HCs (Cuesta et al., 2015). A possible explanation of such incongruence could be found in differences in both the clinical features of the samples and the functional connectivity metric adopted. Only very few MEG studies describe PLI-based networks in AD and MCI. Interestingly, in these articles alterations of global network parameters have been reported in AD but not in MCI. Stam et al. (2009) found that in AD, in a no-task, eyes-closed condition, the clustering coefficient and path length in the lower alpha band were both decreased. Furthermore, Yu et al. (2016), using EEG, found that in the alpha band the global topological features suggested a less integrated topology in AD as compared to controls. Recently, López et al. (2017) focused on brain networks specifically in MCI and found no differences between MCI and controls in terms of global network parameters (averaged normalized weighted clustering coefficient and path length). Our results, obtained with the bias-free MST analysis, are in line with this evidence, indicating that there are no global topological changes in aMCI. Hence, one may speculate that in the very early phase of disease regional changes in functional connectivity (and network topology—see below) may precede global alterations. However, longitudinal studies are needed to confirm this hypothesis.

Comparing the nodal network parameters between groups, we found significant differences in the TPs, in the theta band. The theta rhythm has been linked to memory integration, from the encoding of new information to the retrieval of stored items (for reviews see Kirk and Mackay, 2003; Lisman, 2005). It has been shown that theta power in the medial temporal lobe increases when new memories are included in an existing mnemonic representation (Backus et al., 2016). To this regard, an association between the theta rhythm, AD, and cognitive impairment has been described previously. In fact, higher EEG theta amplitude in parietal, occipital, temporal and limbic areas has been found in AD (Babiloni et al., 2006) and an increased theta relative power has been described using MEG in MCI patients (López et al., 2014), as well as in early AD in almost all cortical regions, including the hippocampi (Engels et al., 2016). Such evidence shows that the theta band yields information that is specifically relevant for AD and MCI.

Our data show changes in topological features in the TPs. More specifically, in the left superior TP, degree and BC were significantly lower in aMCI as compared to controls, while in the right middle TP the BC was higher in the aMCI group. BC and degree are both indices of node centrality in the network. The BC reflects the importance of a node for the interactions among the other nodes in the network, and the degree reflects how many connections insist upon a given node. Our results suggest that the functional role of the TPs is modified in aMCI. Such areas are primarily affected in AD (Delacourte et al., 1999; Thompson et al., 2003). Interestingly, the TPs display a central role within memory circuitry, connecting it to further anatomical structures within and beyond the temporal lobe (Olson et al., 2007).

Interestingly, the nodal features of the left and right TPs display an opposite tendency. This result is partially in agreement with López et al. (2017). They found a higher BC in the lower alpha band in the superior and middle gyrus of the right TP, associated with a decreased BC in upper alpha band in the left middle temporal gyrus. However, none of these findings persisted after FDR correction. The role of the TPs and their functional lateralization has been described as “enigmatic” (Olson et al., 2007). The left TP is mainly considered a semantic hub (Clark et al., 2007), implicated in semantic memory, typically affected in semantic dementia (Landin-Romero et al., 2016). The right TP, given its connections with the amygdala and the orbitofrontal cortex, is involved in emotional control (Chan et al., 2009). By integrating hippocampal information with those from posterior associative areas, the right TP retrieves past emotional experiences to evaluate the meaning of the current stimulus, driving the associated behavioral response (Lane et al., 1999; Nadal, 2013).

Our observation of a “mirrored” change between left and right TP does not find a univocal interpretation. A recent functional connectivity study between the left and right anterior temporal lobe underlined the interaction between the two poles (Warren et al., 2009). The authors proposed that the inhibition of the injured hemisphere by the intact one could be an adaptive process after stroke. The opposite characteristics that we observed in the two TPs could be related to a similar mechanism, in which one TP is influenced by the activity of the contralateral one. Alternatively, it is possible that the neurodegenerative process affects the two TPs at different times. It has been proposed that the increased activity and connectivity often recorded in the early phases of the pathological course may be related to a loss of inhibitory connections, subsequently leading to further neuronal damage, loss of connectivity, and network disruption (de Haan et al., 2012). In this line of thinking, the different alterations observed in the TPs in the right and left hemisphere would be due to differences in the staging of the pathological process.

Finally and interestingly, we observed that, in patients, the BC of the left superior TP showed a negative correlation with the FCSRT delayed total recall. Patients with higher BC show worse memory performances, or vice versa, patients with lower BC have better memory performances. However, it is not clear what the mechanisms that underlie this phenomenon could be. One might speculate that, when the brain damage is still limited, it might be possible to guarantee long term memory efficiency by lowering the computational load in memory circuitry. When the brain damage is widespread, this mechanism would be no longer efficient and the memory performances would worsen. Hence, the inverse relationship between BC and cognitive scores might be due to overload of the TP related to more widespread brain damage. Recently, de Haan et al. (2012) showed that more central areas tend more easily to metabolic overload and consequent degeneration. Probably, the negative relationship observed between BC and memory performance could be explained within this framework.

Comparing the PLI values of the links that connect the right middle TP and the left superior TP with other brain regions, the right middle TP appeared to be more weakly connected to the right cuneus in the aMCI group as compared to the control group. This finding is in line with recent evidence showing that posterior links are hypoconnected in MCI as compared to controls, while the opposite happens for anterior links (López-Sanz et al., 2017).

In conclusion, we aimed to identify changes in network properties that could characterize aMCI, which may precede the development of overt AD (Shah et al., 2000; Petersen et al., 2009). Our results indicate that in aMCI there are changes in network centrality with an opposite trend between left and right TP, with no concurrent global network alteration. These changes are frequency-specific in the theta band, which is classically associated with memory processes. Our findings suggest that the pathological process induces changes in the role of the TP within the functional brain networks, and that the extent of these changes is related to the memory performance in aMCI patients.

## Author Contributions

FJ collected the sample, performed the neuropsychological assessment, performed the MEG recordings, pre-processed the MEG data, wrote the manuscript and prepared the figures. PS collected the sample, performed the MEG recordings, pre-processed the MEG data, performed topological and statistical analysis and wrote the manuscript. AL performed the neuropsychological assessment, performed the MEG recordings and pre-processed the MEG data. RR performed the MEG recordings, pre-processed the MEG data, and contributed to MEG data analysis and statistical analysis. FB collaborated with the MEG data analysis and with the statistical analysis. CC and MA collaborated with the MRI data analysis. MO performed the MRI recordings. AI, VM and AC collected the sample. CG supervised the study. AH contributed to interpreting the results and critically revised the article. GS supervised the study and wrote the manuscript. All authors read and approved the final version of the manuscript.

## Conflict of Interest Statement

The authors declare that the research was conducted in the absence of any commercial or financial relationships that could be construed as a potential conflict of interest.

## Footnotes

^1^http://imaging.mrc-cbu.cam.ac.uk/imaging/Templates

## Abbreviations

BC: betweenness centrality
MEG: magnetoencephalography
MST: minimum spanning tree
PLI: phase lag index
TP: temporal pole.
